# Researchers’ perceptions of ethical challenges in cluster randomized trials: a qualitative analysis

**DOI:** 10.1186/1745-6215-14-1

**Published:** 2013-01-03

**Authors:** Andrew D McRae, Carol Bennett, Judith Belle Brown, Charles Weijer, Robert Boruch, Jamie Brehaut, Shazia Chaudhry, Allan Donner, Martin Eccles, Jeremy Grimshaw, Merrick Zwarenstein, Monica Taljaard

**Affiliations:** 1 Division of Emergency Medicine, University of Calgary, Foothills Medical Centre, Rm C231, 1403 – 29 Street NW, Calgary, Alberta T2N 2T9, Canada; 2 Clinical Epidemiology Program, Ottawa Hospital Research Institute, Ottawa, ON, Canada; 3 Centre for Studies in Family Medicine, Department of Family Medicine, The University of Western Ontario, London, Ontario, Canada; 4 Department of Philosophy, Philosophy and Medicine, Rotman Institute of Philosophy, University of Western Ontario, London, Ontario, Canada; 5 Education and Statistics, Graduate School of Education, University of Pennsylvania, Philadelphia, PA, USA; 6 Ottawa Hospital Research Institute, Clinical Epidemiology Program, Ottawa Hospital, Ottawa, Ontario, Canada; 7 Ottawa Hospital Research Institute, Clinical Epidemiology Program, Ottawa, Ontario, Canada; 8 Epidemiology and Biostatistics, Schulich School of Medicine and Dentistry, The University of Western Ontario, London, Canada; 9 Clinical Effectiveness, Institute of Health & Society, Newcastle University, Newcastle upon Tyne, UK; 10 Ottawa Hospital Research Institute Department of Medicine, University of Ottawa, Ottawa, ON, Canada; 11 Institute for Clinical Evaluative Sciences, Toronto, Ontario, Canada; 12 Department of Epidemiology and Community Medicine, University of Ottawa, Ottawa, Canada

**Keywords:** Cluster randomized trials, Research ethics, Informed consent, Clinical trials, Bioethics, Knowledge translation, Quality improvement, Implementation research

## Abstract

**Background:**

Cluster randomized trials (CRTs) pose ethical challenges for investigators and ethics committees. This study describes the views and experiences of CRT researchers with respect to: (1) ethical challenges in CRTs; (2) the ethics review process for CRTs; and (3) the need for comprehensive ethics guidelines for CRTs.

**Methods:**

Descriptive qualitative analysis of interviews conducted with a purposive sample of 20 experienced CRT researchers.

**Results:**

Informants expressed concern over the potential for bias that may result from requirements to obtain informed consent from research participants in CRTs. Informants suggested that the need for informed consent ought to be related to the type of intervention under study in a CRT. Informants rarely expressed concern regarding risks to research participants in CRTs, other than risks to privacy. Important issues identified in the research ethics literature, including fair subject selection and other justice issues, were not mentioned by informants. The ethics review process has had positive and negative impacts on CRT conduct. Informants stated that variability in ethics review between jurisdictions, and increasingly stringent ethics review in recent years, have hampered their ability to conduct CRTs. Many informants said that comprehensive ethics guidelines for CRTs would be helpful to researchers and research ethics committees.

**Conclusions:**

Informants identified key ethical challenges in the conduct of CRTs, specifically relating to identifying subjects, seeking informed consent, and the use of gatekeepers. These data have since been used to identify topics for in-depth ethical analysis and to guide the development of comprehensive ethics guidelines for CRTs.

## Background

Cluster randomization is a research design commonly used in public health, educational, social science and health services, and implementation research [1]. In cluster randomized trials (CRTs), intact groups or clusters of individuals (for example, medical practices, schools, or communities) are randomly assigned to different intervention arms. The effectiveness of the study intervention is then evaluated using data collected from individual cluster members, often extracted from clinical records or taken from linked, anonymized administrative databases [1]. CRTs may be contrasted with conventional randomized controlled trials, in which individual research participants are randomly assigned to different intervention arms and the effectiveness of the study intervention is evaluated using data collected from each participant.

Because of their distinct methodological features, CRTs pose unique ethical challenges. For example, it can be difficult to identify precisely who is the research subject in a CRT, particularly in large community-based public health CRTs, and in CRTs evaluating educational interventions aimed at health professionals [2,3]. It can be challenging to evaluate risks and potential benefits when the units of randomization, intervention, and outcome measurement are different [4,5]. It is unclear under which circumstances, and from whom, informed consent is required in large community-based CRTs [1,4,6]. Some CRTs have employed ‘gatekeepers’, that is, individuals who have made decisions regarding CRT participation on behalf of randomized clusters [7].

Here we report on a qualitative analysis of interviews with experienced CRT investigators with the objective of documenting ethical challenges encountered in the conduct of CRTs. The objectives of this study were to document, using a descriptive qualitative approach [8-11], the views and experiences of CRT researchers with respect to: (1) ethical issues that have arisen in CRTs and how they have been addressed in practice; (2) the ethics review process for CRTs; and (3) the need for comprehensive ethics guidelines for CRTs. These informants were chosen because of their experience in conducting CRTs, and familiarity with the ethical challenges associated with this study design.

This study was performed as a preparatory step in the development of comprehensive guidelines for the ethical design and conduct of CRTs [12,13]. The findings of this investigation were used to identify key topic areas for in-depth ethical analysis [14-17], as well as to help identify ethical challenges to be addressed during the development of *The Ottawa Statement on the Ethical Design and Conduct of Cluster Randomized Trials*[13].

## Methods

This study was approved by the Ottawa Hospital Research Ethics Board (File 2007191-01H) and the University of Western Ontario Health Sciences Research Ethics Board (File 13755E).

### Sample recruitment

A purposive sample of English-speaking potential informants was identified by senior members of the study team [10] and selected because of their experience in conducing CRTs in various fields. To be considered eligible, potential informants must have been the primary investigator on two or more CRTs or have published papers addressing the ethical challenges in CRTs. These inclusion criteria and sample of potential informants were chosen to ensure that informants would be familiar with the ethical issues in CRTs.

Initial contact with potential informants was made via email by senior members of the study team (RB, AD, ME, JG, MZ). The email introduced the study design and purpose, and inquired about the potential informant’s willingness to participate. If the potential informant was willing to participate, the interviewer (AM, CB) arranged a time for the telephone interview. A letter of information and a copy of the interview guide were sent by email. At the time of the interview, informants were notified that the interview would be recorded and transcribed, but that no identifiable features would be reported. Verbal consent for participation was obtained, and the interview was conducted.

### Final sample

The target sample size in qualitative research is achieved when data saturation occurs, that is when no new themes are identified with respect to a particular question of interest in successive interviews. Twenty-five potential informants were approached to participate in the study. Four individuals declined to participate. The interview from one informant was discarded as the recording was of insufficient quality for transcription and analysis. After analyzing 20 transcribed interviews, data saturation with respect to responses around the issue of informed consent in CRTs had been achieved.

The final sample included 20 experienced CRT researchers. There were 10 participants based in Europe, six based in the USA, and four based in Canada. Five respondents self-identified as statisticians, while the remainder described themselves as researchers. Eleven respondents were in the primary care field, six in hospital-based care or health services, and three in public health. All participants had been co-investigators on between two and twenty CRTs.

### Data collection

A semi-structured telephone interview guide was developed and pilot tested on colleagues. The interview guide included questions about informants’ experience with CRTs, ethical issues in CRTs, ethical challenges encountered with particular CRTs and the ethics review process, and questions seeking input on the need for ethics guidelines for CRTs. Two trained interviewers (AM and CB) conducted the interviews in English. The interview guide was modified in real-time by the interviewers to allow them to seek clarification from the informants or to probe important issues raised by the informants. The interview guide was also updated in an iterative fashion to explore issues that were raised by informants in previous interviews. Trustworthiness and credibility of the data was ensured by the following: verbatim transcripts of the key informant interviews, independent and team analysis, field notes generated after each interview, and reflection and discussion of biases and beliefs which could influence the researchers’ interpretation of the data.

### Data analysis

Each interview transcript was imported into qualitative data analysis software (NVivo 8, QSR Inc.). A directed content analysis approach was used, in that predetermined text analysis categories were used [8-11]. The initial coding template for response categorization was developed by consensus of the investigators. Each transcript was reviewed independently by two investigators (AM and CB), and responses assigned to the appropriate thematic coding categories; new categories were added in an iterative fashion by each researcher to his or her coding template in order to accommodate response themes not corresponding to the predetermined coding themes.

After the initial coding, the two reviewing investigators met and resolved disagreements in the emerging coding assignments by consensus. The coding template was then revised to include additional categories created in the first round of independent analysis, and to delete unused categories. This template revision also ensured fidelity in a second round of thematic coding, as both reviewing investigators were working from the same revised coding template. A second round of thematic coding was performed by the two reviewing investigators, using the new master coding template. Following this, the reviewing investigators met again to resolve discrepancies by consensus.

## Results

Informants responded to questions addressing main pre-identified categories of: (1) ethical issues in CRTs; (2) experiences with the ethics review process for CRTs; and (3) the need for, and input on, possible ethics guidelines for CRTs. Responses were categorized into sub-headings that best described the issues addressed by informants.

### Ethical issues in CRTs

#### The need for informed consent

Informants’ answers to the question of ‘In which circumstances is informed consent required from individual research participants?’ varied widely. For many informants, whether or not informed consent was required in a particular CRT depended on the kind of intervention being evaluated, specifically on whether it was an individual clinical treatment or a health service or other cluster level intervention.

‘The type of intervention that is being trialed is… of crucial importance. So, for example, whether… you are changing the way the entire service is delivered, or whether you are intervening at an individual level and you are just randomizing at a higher level for convenience… it is about which level… the intervention [is] being targeted at…’ (Informant 11, Primary Care Researcher)

The type of data collection procedures used in a CRT could also determine the need for consent from cluster members. Researchers almost universally obtained consent from cluster members if they interacted with these cluster members or intervened upon them to collect data.

‘If we interact with the participant we get consent first. If we are making observations in a public setting, we are not required to get consent… Those activities don’t require consent.’ (Informant 3, Public Health Researcher)

Many informants related concerns over the effect of obtaining informed consent on the validity of a CRT’s findings. In particular, informants worried that disclosure of the nature of the interventions under study in CRTs of behavioral interventions may lead to bias if research participants modify their behavior as a result of information disclosed during consent negotiations rather than as a result of the study interventions.

‘One of the things I am concerned about is bias. If you get really informed consent from people in trials it results in either bias or contamination.’ (Informant 5, Statistician)

With respect to studies of health services or quality improvement interventions, in which the only involvement of individual patients is the use of their health information for data collection, informants expressed concern that requiring informed consent may make such CRTs logistically unfeasible. Several informants felt that these methodological challenges were sufficient to justify a waiver of informed consent for cluster members.

‘Wherever people propose that [requiring individual patient consent], it is the death of those kinds of studies. It is the death of health services research. You can quote me on that. If you require consent to use the data… to look at the performance of a system, it will be a complete disaster.’ (Informant 1, Hospital Care/ Quality Improvement (QI) Researcher)

With respect to CRTs in healthcare implementation research, informants had different perspectives on when, if ever, consent or permission from healthcare professionals is required. Investigators often asked permission, either from individual healthcare professionals or from a group practice, to enrol these professionals in their study.

‘Normally [permission would be obtained] at a general practice level. That would have been done at a partner level, so there would be a discussion within the practice and then agreement at a practice level. There would have been consensual agreement between the partners, between the individual general practitioners that their practice would take part.’ (Informant 11, Primary Care Researcher)

Other informants proceeded with practice-based CRTs without securing the agreement of all healthcare professionals in the practice who might be affected by the intervention.

‘For the ones targeted at practitioners, we had to install software in their electronic medical records systems and computers in their offices so that it would have been GPs in the practice who gave consent. Within a practice, they didn’t all have to agree.’ (Informant 2, Primary Care Researcher)

Some informants argued that healthcare professionals have a professional obligation to participate in CRTs involving a knowledge translation or quality improvement intervention, which therefore overrides any requirement to obtain consent.

‘I would argue that [there is a] professional responsibility to practitioners to take part in research that involves clinical knowledge.’ (Informant 9, Primary Care Researcher)

### Role of the cluster gatekeeper or decision-maker

Informants identified several ethical challenges related to the role of the gatekeeper: the individual who makes a decision with respect to CRT participation on behalf of a cluster. Informants noted challenges identifying the appropriate gatekeeper for certain kinds of groups, particularly municipalities. Opinions varied on whether municipal leaders had the appropriate authority to allow their community to participate in a CRT.

‘In some instances there really is no party to go to for permission when we are doing a community study for example and we are randomly assigning counties or cities. There really isn’t anybody that gives permission for that kind of thing. Even in a city where there is a mayor, the mayor can’t give permission for a city to participate in something. At least that has always been my view.’ (Informant 3, Public Health Researcher)

Informants also recognized that some clusters, such as schools and hospitals, may have multiple gatekeepers because of the organizational structure of these institutions.

‘First off you have to have the district agree that you can even work in this district. Then you have to get the principal to agree that they want to participate in the project. And then we had… the president of the local parent leadership group.’ (Informant 16, Public Health Researcher)

In situations in which researchers had difficulty identifying the appropriate gatekeeper for community-based research, they typically sought the approval of some local advisory committee.

‘Our approach in virtually every instance was to organize a local community advisory board made up of community residents in the city if we were working with cities or in the county if we were working with counties. We would get their input on a variety of things though the basic design was set.’ (Informant 3, Public Health Researcher)

Another ethical challenge concerning gatekeepers related to the scope of the gatekeeper’s decision-making authority. Responses varied on whether the gatekeeper possessed sufficient authority to provide consent on behalf of all cluster members, or whether the gatekeeper was simply permitting access to individual cluster members who would subsequently provide consent for CRT participation.

‘I think the main issues for me still stem around the issue of consent. Who [gives] consent? Whether consent needs to be achieved at every level of cluster or whether almost guardian consent is acceptable and that has been where the most discussion has happened really about in the ethical issues of cluster trials for me.’ (Informant 11, Primary Care Researcher)

Some informants seemed comfortable accepting a gatekeeper’s consent on behalf of an entire cluster for studies that were evaluating cluster-level interventions, such as educational or quality improvement interventions targeted at healthcare systems or practitioners.

‘I would say most of them have been looking towards a cluster guardian… consent because most of the interventions I have been involved in have been mainly around management interventions where the real intervention is at the level of either the practitioner, the health professional or the healthcare organization and this specific intervention hasn’t really been targeted at the lower level of the cluster, the patient level.’ (Informant 11, Primary Care Researcher)

Other informants expressed a different opinion. They felt it was important to obtain consent from individual patients in healthcare CRTs, regardless of whether consent for cluster enrolment was obtained from a gatekeeper.

‘So there is… consent for the patient and… consent for, in our case, the practice, so there are two levels of consent if you like. If there wasn’t patient consent involved, then obviously there would be very significant ethical issues but I have come to the view that if patients are given information and they consent, then that is fine.’ (Informant 9, Primary Care Researcher)

### Risks and potential benefits

Our interview guide included items addressing the risks posed by CRT participation. Informants had few concerns regarding the risks posed to cluster members by CRTs.

‘Risks were none. I think we came up with some for the ethics committee.’ (Informant 14, Primary Care Researcher)

The interventions under evaluation in CRTs were commonly perceived as standard care with little or no incremental risk to cluster members.

‘None of the interventions that we have evaluated have put anyone at any kind of risk… there is certainly little risk involved.’ (Informant 3, Public Health Researcher)

For healthcare CRTs that evaluated the effect of interventions on health professionals using patients’ health information, informants identified threats to privacy as the sole risk.

‘I think the core risk is loss of privacy. That really is the only issue because we weren’t studying… a therapeutic intervention.’ (Informant 8, Hospital Care/QI Researcher)

Some informants voiced concern that members of clusters assigned to control groups may not benefit from an experimental intervention.

‘The dilemma and the tension again was this trial that is basically about the QI where the controlled practices got nothing. They didn’t get anything but normal care.’ (Informant 14, Primary Care Researcher)

One commonly employed solution to address this dilemma was to offer the experimental intervention to the control clusters after the CRT had been completed.

‘Sometimes we are so concerned about the control arm feeling that they don’t get anything that it might affect recruitment, that we offer them the intervention once the trial is over.’ (Informant 14, Primary Care Researcher)

An additional risk identified by one informant was that CRT enrolment may entail an increased clinical or administrative workload for participating medical practices.

‘The only risk I feel in doing a lot of research here is practices become overburdened by having to do research.’ (Informant 5, Statistician)

#### Experiences with the ethics review process

Many of the informants noted wide variability among research ethics committees, and across jurisdictions, in both the ethics review process and in ethics committees’ decisions. Informants noted that this variability has made it more difficult to do multicenter CRTs.

‘Cluster… trials of hospitals randomize independent institutions, each of which has a [research ethics committee]. Each [research ethics committee], with its slightly different application procedures, forms, and timelines has been a separate and trying process.’ (Informant 7, Primary Care Researcher)

Several informants commented that the ethics review process was easier in the past, and has become more cumbersome in recent years.

‘… Generally it hasn’t been too bad up until the last 5 years. Beforehand we were quite comfortably able to get ethics approval… but it is different now.’ (Informant 13, Primary Care Researcher)

However, other informants commented that as research ethics committees become familiar with the CRT design, the review process has gone more smoothly.

‘… Now CRTs are widely accepted research methods, particularly in primary care studies, primary care settings. Ethics committees are now actually quite comfortable with them.’ (Informant 14, Primary Care Researcher)

Informants’ opinions varied on the effect of the ethics review process and regulatory requirements on the validity of a CRT. Half of the informants reported a positive impact of the ethics review process on the quality of their studies, while the other half reported negative effects. Perceived negative effects included threats to validity from consent processes, and diminished enrolment because of consent requirements.

‘As the participation rates drop… then the results are less generalizable and less helpful. There is no question that the higher hurdles for consent in school studies and certainly in clinic based studies have made it more difficult to do the work and to get high participation rates.’ (Informant 3, Public Health Researcher)

Perceived positive effects included requirements for greater methodological rigor and thoughtfulness in study design, and improved protections for research participants.

‘I am a great believer that [research] ethics committee[s] do ask… searching questions… Just the process of thinking about the ethical implications of your design is something that we might not do if we didn’t have to go to ethics committees… I think I would say for all my research, it is improved the quality of what we do.’ (Informant 14, Public Health Researcher)

#### Developing ethics guidelines for CRTs

Informants were asked explicitly about the need for guidelines for the ethical conduct of CRTs. Generally, informants were supportive of efforts to develop ethics guidelines for the design, conduct, and review of CRTs, and identified informed consent as an ethical challenge for which investigators and ethics committees are in particular need of guidance.

‘I think there does still need to be a discussion document… on when is individual level consent an absolute requirement.’ (Informant 11, Primary Care Researcher)

When asked for suggestions for the content of ethics guidelines, another common response was to suggest inclusion of educational material for research ethics committees on the ethical and methodological aspects of CRTs that make CRTs distinct from individually randomized trials.

‘I think that there are issues which make cluster trials different to the sort of trials that [research ethics committees] normally see and that it would give me confidence as an investigator if I knew that they fully understood the difference.’ (Informant 20, Hospital Care/QI Researcher)

## Discussion

CRTs have unique ethical issues, primarily because in a CRT the units of randomization, intervention, and outcome measurement may be different. This study was designed to elicit the views of experienced CRT researchers on the key ethical issues involved in the conduct of CRTs, and to describe their experiences with the ethics review process for CRTs. Key issues identified by informants included challenges around when, and from whom, informed consent must be obtained, the role of gatekeepers, and perceived challenges relating to the ethics review processes. Other important ethical issues, such as privacy, study benefits and harms, and justice issues, were rarely mentioned by informants.

Informants’ opinions on whether or not informed consent should be obtained from cluster members appeared to depend on the scientific question and experimental interventions in a particular CRT. One issue often cited as a possible concern in CRTs is the risk of various forms of bias induced by consent practices in CRTs, particularly when it is not possible to seek informed consent prior to randomization. These concerns have previously been noted in the CRT literature [4,18].

Informants reported challenges in identifying an appropriate gatekeeper who has legitimate authority to grant permission for a cluster (such as a municipality or social group) to be enrolled in a CRT, and uncertainty about the scope of a gatekeeper’s authority. A recent paper from this study team has challenged some of the traditional roles of gatekeepers, while also identifying novel functions for gatekeepers in CRTs [7]. Specifically, the role for gatekeepers as substitute decision-makers when individual consent is not possible is limited [7]. However, gatekeepers may legitimately represent cluster-level interests in specific instances [7]. Investigators and research ethics committees should be mindful of the appropriate roles for gatekeepers when considering their use.

Many informants identified the problem of variability in ethics review findings between jurisdictions, which represents a challenge to the successful conduct of multicenter CRTs [19]. They also described a perception of increasingly onerous oversight requirements imposed by ethics committees in recent years, particularly increasingly stringent consent requirements. Variability in research ethics committee decisions may be attributable to differences in national research guidelines and regulations, although some informants noted differences in ethics committee findings within the same country. If broadly adopted, *The Ottawa Statement on the Ethical Design and Conduct of Cluster Randomized Trials*[13] may be helpful in addressing variation in ethics review among research ethics committees while simultaneously clarifying for researchers when informed consent is required and what other subject protections are necessary.

This work represents an example of how empirical studies may be used to address novel challenges in research ethics. Existing ethics guidelines did not adequately address some of the ethical challenges stemming from the CRT design. This led to variability in research ethics review and in the steps taken by researchers to protect subjects’ interests. In order to develop comprehensive ethics guidelines for CRTs, it was important to identify which ethical challenges posed difficulties for researchers and research ethics committees in practice.

Challenges relating to the identification of subjects and informed consent and the role of gatekeepers have been addressed with in-depth ethical analysis in a series of papers from this research team [14-17]. Informants’ responses have also provided a list of key topics were used in the development of the *Ottawa Statement on the Ethical Design and Conduct of Cluster Randomized Trials*[13].

### Limitations

Our sample included experienced CRT researchers who have contributed to the literature on the ethics of CRTs. Our informants were English-speaking researchers who have worked mostly in developed countries, although some had performed CRTs in developing countries. Their experiences may not necessarily be transferable to all CRT researchers. However, we are reassured that experienced researchers from a variety of geographic locations, and in a variety of research fields, voiced similar opinions on key ethical issues. Furthermore, the extensive experience of the informants interviewed in this study lends weight to their views, and to our conclusions.

## Conclusion

Informants described perceived challenges with the ethics review process, expressed concern over when informed consent is required from cluster members, and over the authority of cluster gatekeepers. Other important ethical challenges, such as the relationship between harms and benefits and issues of distributive justice were not highlighted by informants. Subsequent papers published by this research team, as well as the *Ottawa Statement on the Ethical Conduct and Review of Cluster Randomized Trials*[13] aim to address these challenges.

## Competing interests

The authors declare they have no competing interest.

## Authors’ contributions

AM helped conceive the study, contributed to the design of the interview template, designed the analytic approach, conducted the interviews, analyzed the data, drafted the manuscript, and approved the final version. CB conducted the interviews, analyzed the data, contributed to revisions of the manuscript, and approved the final version. JBB assisted with the design of the interview template and analytic approach, contributed to manuscript revisions, and approved the final version. CW contributed to the design of the study and interview template, contributed to revisions of the manuscript, and approved the final version. RB contributed to revisions of the manuscript and approved the final version. JB contributed to revisions of the manuscript and approved the final version. SC contributed to revisions of the manuscript and approved the final version. AD contributed to the design of the study, recruitment of participants, revision of the manuscript, and approved the final version. ME contributed to the design of the study, recruitment of participants, revision of the manuscript, and approved the final version. JG contributed to the design of the study, recruitment of participants, revision of the manuscript, and approved the final version. MZ contributed to the design of the study, recruitment of participants, revision of the manuscript, and approved the final version. MT designed the study, contributed to the design of the interview template and analytic approach, revisions of the manuscript, and approved the final version.
